# Erfassung der Labortestungen auf SARS-CoV-2 in Deutschland

**DOI:** 10.1007/s00103-021-03307-y

**Published:** 2021-04-15

**Authors:** Janna Seifried, Alexandra Hoffmann, Sarah Reda, Sindy Böttcher, Muna Abu Sin, Alexandra Hofmann, Ines Noll, Anja von Laer, Annicka Reuss, Djin-Ye Oh, Stefan Albrecht, Daniel Stern, Niklas Willrich, Doreen Staat, Benedikt Zacher, Marc Schneider, Marcel Feig, Andreas Nitsche, Thorsten Rieck, Ute Rexroth, Tim Eckmanns, Osamah Hamouda

**Affiliations:** 1 Abteilung für Infektionsepidemiologie, Robert Koch-Institut, Seestraße 10, 13353 Berlin, Deutschland; 2 Abteilung für Infektionskrankheiten, Robert Koch-Institut, Berlin, Deutschland; 3 Abteilung für Epidemiologie und Gesundheitsmonitoring, Robert Koch-Institut, Berlin, Deutschland; 4 Zentrum für Biologische Gefahren und Spezielle Pathogene, Robert Koch-Institut, Berlin, Deutschland

**Keywords:** SARS-CoV‑2, Testzahlen, Testkapazitäten, Surveillance, Abrechnungsdaten, SARS-CoV‑2, Test numbers, Test capacities, Surveillance, Billing data

## Abstract

Die Erfassung von Daten zu SARS-CoV-2-Testungen in Deutschland sind für die Einschätzung des Infektionsgeschehens im Rahmen der COVID-19-Pandemie von zentraler Bedeutung. Am Robert Koch-Institut (RKI) werden dazu die Daten aus verschiedenen Systemen zur Erfassung von Labortestungen zusammengeführt. In diesem Beitrag werden zunächst bedeutsame Aspekte der Testverfahren erläutert. Nachfolgend werden die unterschiedlichen Systeme zur Erfassung von Labortestungen erläutert und Testzahlen aus der RKI-Testlaborabfrage und der laborbasierten Surveillance SARS-CoV‑2 sowie die Abrechnungsdaten der kassenärztlichen Vereinigungen zu SARS-CoV-2-Labortestungen dargestellt.

Mit der RKI-Testlaborabfrage und der laborbasierten Surveillance SARS-CoV‑2 stand früh in der Pandemie eine Surveillance zur Verfügung, mit der unter den teilnehmenden Laboren Daten zu durchgeführten Testungen und Testkapazitäten ausgewertet werden können. Durch die Erfassung von positiven und negativen Testergebnissen sind Aussagen zur Gesamtzahl der durchgeführten Testungen sowie dem Anteil der positiven Testergebnisse möglich. Während die aggregierten Testzahlen bundesweit weitestgehend repräsentativ sind, ist die Repräsentativität auf Bundesland- und Landkreisebene aber nicht immer gegeben. Die Abrechnungsdaten der Kassenärztlichen Vereinigungen können die Labordaten im Nachhinein ergänzen. Sie erlauben eine retrospektive Einschätzung der Gesamtzahl von SARS-CoV-2-Tests in Deutschland, da die Leistungen der vertragsärztlichen Versorgung aller gesetzlich Krankenversicherten (ca. 85 % der Bevölkerung) enthalten sind.

Die verschiedenen Systeme zur Erfassung von Labortestungen ergänzen sich gegenseitig. Die Auswertungen fließen in die Maßnahmenempfehlungen zur Pandemiebewältigung ein.

## Abstract (en)

The collection of data on SARS-CoV‑2 tests is central to the assessment of the infection rate in the context of the COVID-19 pandemic. At the Robert Koch Institute (RKI), data collected from various laboratory data recording systems are consolidated. First, this article aims to exemplify significant aspects regarding test procedures. Subsequently the different systems for recording laboratory tests are described and test numbers from the RKI test laboratory query and the laboratory-based SARS-CoV‑2 surveillance as well as accounting data from the Association of Statutory Health Insurance Physicians for SARS-CoV‑2 laboratory tests are shown.

Early in the pandemic, the RKI test laboratory query and the laboratory-based SARS-CoV‑2 surveillance became available and able to evaluate data on performed tests and test capacities. By recording the positive and negative test results, statements about the total number of tests and the proportion of positive test rates can be made. While the aggregate test numbers are largely representative nationwide, they are not always representative at the state and district level. The billing data of the Association of Statutory Health Insurance Physicians can complement the laboratory data afterwards. In addition, it can provide a retrospective assessment of the total number of SARS-CoV‑2 numbers in Germany, because the services provided by statutory health insurers (around 85% of the population in Germany) are included. The various laboratory data recording systems complement one another and the evaluations flow into the recommended measures for the pandemic response.

## Einleitung

Die Erfassung von SARS-CoV-2-Testzahlen ist für die Einschätzung des Infektionsgeschehens im Rahmen der COVID-19-Pandemie zentral. Die Labordiagnostik spielt hierbei nicht nur eine wesentliche Rolle in der diagnostischen Abklärung von Verdachtsfällen, sondern auch für die Beurteilung der epidemiologischen Entwicklung und hinsichtlich Strategien zur Verlangsamung des aktuellen Geschehens in Deutschland [1, 2].

Nach dem Infektionsschutzgesetz (IfSG) sind bisher nur Fälle mit einer labordiagnostisch nachgewiesenen Infektion (direkter Erregernachweis, z. B. PCR, Virusanzucht oder Antigennachweis) meldepflichtig. Dies allein lässt keine Rückschlüsse auf die Gesamtzahl der Testungen zu. Die Gesamtzahl an durchgeführten Tests beeinflusst jedoch die Anzahl der täglich gemeldeten COVID-19-Fälle: Eine größere Anzahl an positiven Testungen führt in der Regel zu einer vollständigeren Erfassung von Erkrankungsfällen. Für eine genauere Einschätzung eines tatsächlichen Anstiegs von Infizierten ist neben der absoluten Anzahl insbesondere der Anteil positiv Getesteter innerhalb der Gesamtzahl von Testungen ausschlaggebend. Eine Meldepflicht, die die negativen Testergebnisse miteinschließt, würde eine vollerfassende Dokumentation der Testsituation in Deutschland ermöglichen.

Mit Beginn der Pandemie mussten zum einen rasch diagnostische Verfahren zum Nachweis von SARS-CoV‑2 in den Laboren etabliert werden. Zum anderen ist es für die Beurteilung der epidemiologischen Lage und Steuerung des Geschehens unerlässlich, Daten zu Testkapazitäten sowie differenzierte Daten zu Testungen und Testergebnissen zu erfassen. Am Robert Koch-Institut (RKI) wurden zeitnah neue Instrumente zur Datenerhebung geschaffen, bestehende Surveillance-Systeme ausgebaut und weitere Datenquellen genutzt, um entsprechend der hohen Dynamik des Geschehens rasch Aussagen über das Testgeschehen machen zu können. Es ist der engagierten Mitarbeit der Labore zu verdanken, dass Daten zu Testungen in Deutschland über verschiedene Übermittlungsformen zur Verfügung stehen.

Im Folgenden werden für die epidemiologische Bewertung bedeutsame Aspekte der Diagnostik zum Nachweis von SARS-CoV‑2 sowie die Systeme zur Erfassung der Testzahlen vorgestellt und diskutiert.

## Diagnostik

### Nachweisverfahren von SARS-CoV-2

Für eine labordiagnostische Untersuchung zur Klärung des Verdachts auf eine SARS-CoV-2-Infektion stehen für die Routinediagnostik in Deutschland 2 unterschiedliche Testverfahren für den direkten Erregernachweis zur Verfügung: PCR-Methoden mittels Nukleinsäureamplifikationstechnik (NAAT) und Antigentests. Der Nachweis von SARS-CoV‑2 mittels Reverse-Transkriptase-Polymerase-Kettenreaktion (RT-PCR) gilt als Goldstandard und zeichnet sich durch eine sehr hohe Sensitivität und Spezifität aus. Für die Durchführung wird eine entsprechende Laborausstattung und Fachpersonal benötigt, welches nur in begrenztem Umfang zur Verfügung steht.

Antigentests lassen sich mit deutlich weniger Aufwand und Infrastruktur durchführen, weisen jedoch eine geringere Sensitivität und Spezifität auf als die RT-PCR. Daher stellt ein positives Antigentestergebnis zunächst einen Verdacht auf das Vorliegen einer SARS-CoV-2-Infektion dar. Die Diagnosestellung durch die Bestätigung mittels RT-PCR sowie ärztlicher Beurteilung sichert die Qualität der gemeldeten Fallzahlen.

Neben dem molekularbiologischen Nachweis des SARS-CoV-2-Genoms kann für wissenschaftliche Fragestellungen auch eine Virusanzucht durchgeführt werden. Diese ist jedoch Speziallaboren mit entsprechender Schutzstufe vorbehalten (in Deutschland Biosafety Level (BSL) 3).

Zum Nachweis einer vorangegangenen SARS-CoV-2-Infektion stehen zudem verschiedene kommerzielle Testformate (Enzyme-linked Immunosorbent Assay, ELISA; Chemilumineszenz-Immunoassays, CLIA) mit unterschiedlichen Virusantigenen zur Verfügung, mit denen Immunglobulin (Ig)M-, IgA-, IgG- oder Gesamtantikörper nachgewiesen werden können. Das Vorhandensein von neutralisierenden Antikörpern kann mithilfe eines Neutralisationstests nachgewiesen werden, der aber ähnlich wie die Virusanzucht nur in Speziallaboren durchgeführt werden kann. Ersatzverfahren, die auch unter normalen Laborbedingungen durchgeführt werden können, sind mittlerweile verfügbar.

Weitere Informationen zur Indikation und zur Aussagekraft der einzelnen Testverfahren finden sich in den „Hinweisen zur Testung von Patienten auf Infektion mit dem neuartigen Coronavirus SARS-CoV-2“ [3].

### Testungen am Robert Koch-Institut

Im Zentrum für biologische Sicherheit (ZBS1) am Robert Koch-Institut wurden im Januar 2020 die ersten *In-house-*RT-PCR-Assays in 2 unabhängigen Genomregionen zum spezifischen und sensitiven Nachweis von SARS-CoV‑2 etabliert, später wurden verschiedene kommerzielle Systeme ergänzend validiert. Zur Erhöhung des Probendurchsatzes wurden Protokolle für Ansätze wie das Poolen mehrerer Proben und das Multiplexen (Zusammenlegen) der verschiedenen PCR-Systeme in einer Reaktion hinsichtlich der Vor- und Nachteile beschrieben und eingesetzt. Neben der Funktion als Referenzlabor der Weltgesundheitsorganisation (WHO) für SARS-CoV‑2 übernimmt ZBS1 auch wichtige Aufgaben bei der Bereitstellung von Virusstandards, Serumproben für die Standardisierung serologischer Verfahren und Reagenzien. Neben der molekularen Diagnostik von Verdachtsfällen werden auch verschiedene Studien in Zusammenarbeit mit anderen Fachgebieten des RKI bearbeitet.

Von Februar 2020 bis zum 28.02.2021 wurden im Rahmen der molekularen Diagnostik mehr als 85.000 Proben bearbeitet.

Im Rahmen der Virologischen Surveillance am Nationalen Referenzzentrum für Influenzaviren werden Proben aus den in der Arbeitsgemeinschaft Influenza (AGI) angeschlossenen Sentinel-Praxen (ca. 100 ärztliche Praxen) auf verschiedene respiratorische Viren untersucht (Influenzaviren, respiratorisches Synzytialvirus, humanes Metapneumovirus, Parainfluenzaviren sowie Rhino‑/Enteroviren). Seit Februar 2020 werden die Proben zusätzlich auch auf das SARS-CoV‑2 getestet, sodass das Spektrum der für akute Atemwegsbeschwerden verantwortlichen Viren umfassend und differenziert dargestellt wird. Von Februar 2020 bis zum 28.02.2021 wurden im Rahmen des AGI-Sentinels über 6500 Proben untersucht, von denen mehr als 2600 positiv für mindestens einen viralen Erreger waren. SARS-CoV‑2 wurde bei 223 der untersuchten Patienten nachgewiesen. Des Weiteren werden die SARS-CoV-2-Isolate molekularepidemiologisch charakterisiert. Ziel ist hierbei eine umfassende Darstellung der genetischen Diversität in Deutschland zirkulierender SARS-CoV-2-Varianten abhängig von Region und Zeit.

### Sensitivität, Spezifität und die Rolle falsch-positiver Testergebnisse

Es wird häufiger angeführt, dass durch vermehrte ungezielte Testungen der Anteil falsch-positiver Befunde zunimmt. Generell wird die Richtigkeit des Ergebnisses von diagnostischen Tests neben deren Qualitätsmerkmalen und der Qualität von Probennahme, Transport, Durchführung und Befundung auch von der Verbreitung einer Erkrankung/eines Erregers in der Bevölkerung beeinflusst (positiver und negativer Vorhersagewert). Je seltener eine Erkrankung ist und je ungezielter getestet wird, umso höher sind die Anforderungen an die Sensitivität und die Spezifität der zur Anwendung kommenden Tests [4].

Ein falsch-positives Testergebnis bedeutet, dass eine Person ein positives Testergebnis erhält, obwohl keine Infektion mit SARS-CoV‑2 vorliegt. Aufgrund des Funktionsprinzips des PCR-Verfahrens und der hohen Qualitätsanforderungen liegt die analytische Spezifität bei korrekter Durchführung und Bewertung bei nahezu 100 %. Im Rahmen von qualitätssichernden Maßnahmen nehmen diagnostische Labore an Ringversuchen teil. Die bisher erhobenen Ergebnisse spiegeln die sehr gute RT-PCR-Testdurchführung in deutschen Laboren wider [5].

Die bisher verfügbaren, unabhängig erhobenen Studien zu den klinischen Leistungsparametern von Antigentests (www.finddx.org [6], www.diagnosticsglobalhealth.org [7]) deuten auf erhebliche Unterschiede zwischen den kommerziell erhältlichen Antigentests hin, was die Notwendigkeit einer herstellerunabhängigen Validierung zur Ermittlung der Leistungsparameter eines Testes unterstreicht [3]. Aspekte des Zusammenhangs von Vortestwahrscheinlichkeit und Leistungsparametern von Antigentests werden in einer Infografik [8] erläutert.

Die Herausgabe eines klinischen Befundes unterliegt einer fachkundigen Validierung und schließt im klinischen Setting die Anamnese und Differenzialdiagnosen ein. In der Regel werden nicht plausible Befunde in der Praxis durch Testwiederholung oder durch zusätzliche Testverfahren bestätigt bzw. verworfen [3].

Bei korrekter Durchführung der Tests und fachkundiger Beurteilung der Ergebnisse gehen wir demnach von einer sehr geringen Zahl falsch-positiver Befunde aus, die die Einschätzung der Lage nicht verfälscht [9].

## Systeme zur Erfassung von SARS-CoV-2-Testungen in Deutschland

### RKI-Testzahlerfassung

Für die Erfassung der SARS-CoV-2-PCR-Testzahlen werden Daten von Universitätskliniken, Forschungseinrichtungen sowie klinischen und ambulanten Laboren zusammengeführt. Die Erfassung basiert auf einer freiwilligen Mitteilung der Labore und erfolgt über eine webbasierte Plattform (VOXCO, RKI-Testlaborabfrage) in Zusammenarbeit mit der am RKI etablierten Virologischen Surveillance am Nationalen Referenzzentrum für Influenzaviren und ZBS1 sowie der am RKI etablierten, laborbasierten Surveillance SARS-CoV‑2 (eine Erweiterung der Antibiotika-Resistenz-Surveillance, ARS), dem Netzwerk für respiratorische Viren (RespVir) sowie der Abfrage eines labormedizinischen Berufsverbands. Bei den erhobenen Daten handelt es sich um eine freiwillige und keine verpflichtende Angabe der Labore, sodass eine Vollerfassung der in Deutschland durchgeführten PCR-Tests auf SARS-CoV‑2 zum jetzigen Zeitpunkt nicht vorliegt. Die hier veröffentlichten Daten liefern daher Hinweise zur aktuellen Situation in den Laboren, erlauben aber keine detaillierten oder regionalen Auswertungen sowie direkte Vergleiche mit den gemeldeten Fallzahlen [10].

**Figure 1:**
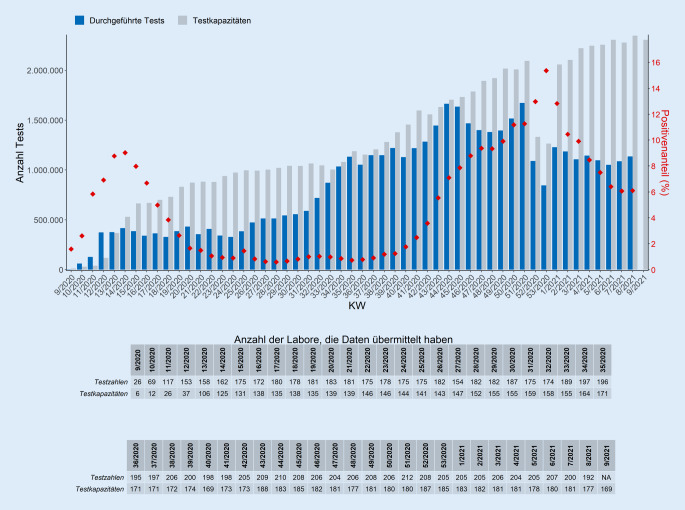

Seit Beginn der Testungen in Deutschland bis einschließlich Kalenderwoche (KW) 8/2021 wurden bisher 45.114.160 PCR-Tests erfasst, davon wurden 2.685.563 positiv auf SARS-CoV‑2 getestet (Datenstand 03.03.2021; Abb. 1).

Bis einschließlich KW 8/2021 haben sich 259 Labore für die RKI-Testlaborabfrage oder in einem der anderen an der Erhebung beteiligten Netzwerke registriert und übermitteln ihre Daten nach Aufruf überwiegend wöchentlich. Da Labore die Tests der vergangenen Kalenderwochen nachmelden können, ist es möglich, dass sich die ermittelten Zahlen nachträglich ändern. Es ist zu beachten, dass die Zahl der Tests nicht mit der Zahl der getesteten Personen gleichzusetzen ist, da in den Angaben Mehrfachtestungen von Patienten enthalten sein können. Daher kann von dem in der Testzahlerfassung angegebenen Positivenanteil auch nicht unmittelbar auf die tatsächliche Prävalenz in der Bevölkerung geschlossen werden. Während die Testaktivität in der Umsetzung der nationalen Teststrategie gut abgebildet wird, sind für eine detaillierte Bewertung der Positivenanteile ergänzende Erfassungssysteme zurate zu ziehen (siehe z. B. die Teilmenge aus der laborbasierten Surveillance SARS-CoV-2).

### Testkapazitäten

Zusätzlich zur Anzahl durchgeführter Tests werden in der RKI-Testlaborabfrage und durch einen labormedizinischen Berufsverband Angaben zur täglichen (aktuellen) Testkapazität erfragt. Diese Angabe ist freiwillig und stellt nur eine Momentaufnahme für die jeweilige Kalenderwoche dar.

In Deutschland ist eine erhebliche PCR-Testkapazität zum qualitätsgesicherten Nachweis von SARS-CoV‑2 vorhanden. Dennoch kann es bei hohem Probenaufkommen zu Lieferengpässen bei Testreagenzien oder Verbrauchsmaterial kommen. Auch das zur Durchführung benötigte Fachpersonal steht nur in begrenztem Umfang zur Verfügung. Die Nationale Teststrategie [11] sieht daher einen zielgerichteten und anlassbezogenen Einsatz der PCR-Tests vor. In KW 8/2021 gaben 169 Labore an, für die kommende KW 9/2021 insgesamt eine theoretische Testkapazität von 2.317.339 PCR-Tests zur Verfügung stellen zu können.

**Figure 2:**
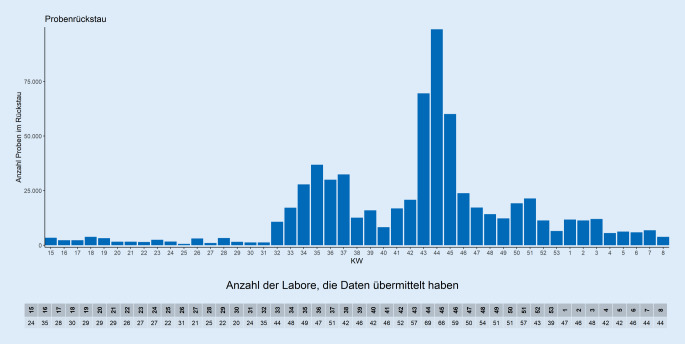

Unter Einbeziehung der Reichweite ergibt sich daraus eine reelle Testkapazität von 2.306.497 Tests in KW 9/2021. Die Reichweite gibt an, an wie vielen Arbeitstagen ein Labor unter Vollauslastung der angegebenen maximalen Testkapazität unter Berücksichtigung aller notwendigen Ressourcen (Personal, Entnahmematerial, Testreagenzien u. a.) zum Zeitpunkt der Abfrage arbeiten kann. Da die Reichweite stark vom Vorhandensein von Testreagenzien abhängig ist, stellt die Angabe eine Momentaufnahme in einem dynamischen System dar. In KW 8/2021 gaben 169 Labore zum Zeitpunkt der Abfrage eine Reichweite von 2–65 Arbeitstagen (Median: 8 Tage) an. Bei sehr hoher Auslastung in den Laboren kann es regional zu einem Rückstau an zu untersuchenden Proben kommen. Dies war in den Wochen um KW 44/2020, in der 69 Labore einen Probenrückstau von knapp 100.000 Proben meldeten, der Fall. Abb. 1 zeigt die erfassten wöchentlichen SARS-CoV-2-PCR-Testzahlen und jeweilige durchschnittliche Positivenanteile der Testungen im Verhältnis zu den übermittelten Testkapazitäten [10], in Abb. 2 wird der Probenrückstau dargestellt.

### Stratifizierte Auswertungen aus der laborbasierten Surveillance SARS-CoV-2

Die Antibiotika-Resistenz-Surveillance (ARS) ist nach § 13 IfSG als laborgestütztes und am RKI angesiedeltes Surveillance-System zur kontinuierlichen Erhebung von Daten der mikrobiologischen Routine für das gesamte Spektrum klinisch relevanter Erreger, einschließlich Viren, konzipiert. Teilnehmer bei ARS sind Labore, die Proben aus medizinischen Versorgungseinrichtungen und Arztpraxen mikrobiologisch untersuchen und auf freiwilliger Basis ihre Daten an das RKI übermitteln. Somit handelt es sich um keine Vollerfassung.

Im Rahmen der SARS-CoV-2-Pandemie konnte die ARS unter Nutzung bereits bestehender Schnittstellen zur elektronischen Datenübermittlung kurzfristig und flexibel genutzt werden, um eine laborbasierte Surveillance SARS-CoV‑2 aufzubauen. Damit wurde eine Datengrundlage sowohl zur weiteren Beurteilung der epidemiologischen Lage in Deutschland als auch zur Bearbeitung wissenschaftlicher Fragestellungen geschaffen.

Es waren wenige Veränderungen und Erweiterungen notwendig, um tagesaktuell für die laborbasierte Surveillance SARS-CoV‑2 relevante Variablen zu erfassen und zu verarbeiten. Durch Übermittlung von pseudonymisierten Falldaten können die Auswertungen unter Ausschluss von Mehrfachtestungen durchgeführt werden. Neben den für die epidemiologische Beurteilung wichtigen Variablen wie Alter und Geschlecht der Patienten, die regionale Zuordnung der Einsender, der Typ der Einsender (Krankenhaus, Arztpraxis), der Fachbereich der Einsender (Fachbereich Arztpraxis bzw. Fachbereich im Krankenhaus) und bei Krankenhäusern der Stationstyp (Normalstation, Intensivstation, Ambulanz etc.), das Entnahme- und Testdatum der Probe sowie das Testergebnis (Erfassung negativer und positiver Ergebnisse) werden auch für die Surveillance SARS-CoV‑2 spezifische Variablen erfasst. Hierzu gehören die neue Sammelkategorie für Einsender wie Drive-in-Stationen, Testzentren, fremdeinsendende Labore etc. sowie die Erfassung der Ergebnisse serologischer Diagnostik (Antikörper- und Antigentests) zu SARS-CoV‑2.

Die Datenübermittlung an ARS erfolgt über eine vom RKI definierte XML-Schnittstelle, die von einem kommerziellen Statistiksoftwarehersteller implementiert wird und in den Laboren ansetzt. Um die Reichweite der Surveillance zu erhöhen und Laboren ohne bisherige Teilnahme an ARS eine Teilnahme an der Surveillance SARS-CoV‑2 zu ermöglichen, wurde ein weiterer Übermittlungsweg eingerichtet. Hierbei können für die Surveillance SARS-CoV‑2 relevante Daten auch als csv.- oder txt.-Dateien aus den Laborinformationssystemen exportiert und dann pseudonymisiert und verschlüsselt an das RKI übermittelt werden. Damit liegen 2 Übermittlungswege vor, die prinzipiell allen Laboren die Teilnahme an der Surveillance SARS-CoV‑2 ermöglichen [2].

Für alle Labore liegen durch retrospektive Datenübermittlung unabhängig vom Beginn der Teilnahme an der Surveillance SARS-CoV‑2 Daten ab der ersten Testung im Jahr 2020 vor, sodass die Datenlage nicht durch sukzessiv steigende Teilnehmerzahlen verzerrt wird.

Mit Stand 23.02.2021 nahmen 73 Labore an der laborbasierten Surveillance SARS-CoV‑2 über ARS teil. Es lagen für diesen Zeitraum insgesamt 18.137.668 PCR-Nachweise vor. Sowohl für die PCR-Nachweise als auch für serologische Befunde werden Auswertungen im zeitlichen Verlauf durchgeführt, womit Trends erkennbar und Verlaufsbeurteilungen möglich werden. Stratifizierte Auswertungen werden wöchentlich im Lagebericht [10] sowie in einem eigenen Wochenbericht [12] veröffentlicht.

**Figure 3:**
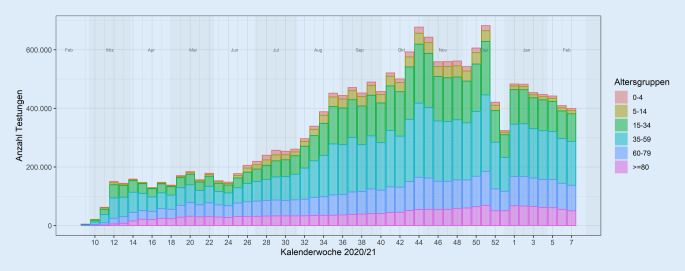

Die laborbasierte Surveillance SARS-CoV‑2 über ARS erfasst sowohl positive als auch negative Testergebnisse, womit Abschätzungen bezüglich der Anzahl der in Deutschland durchgeführten Testungen sowie der Testraten in verschiedenen Strata möglich werden. Abb. 3 zeigt beispielhaft die Anzahl durchgeführter Tests nach Altersgruppe und Kalenderwoche seit den ersten übermittelten Nachweisen von Testungen auf SARS-CoV‑2. Deutlich wird die über den gesamten Zeitraum der Pandemie deutlich höhere Anzahl durchgeführter Testungen in der zweiten Jahreshälfte 2020 im Vergleich zum Jahresanfang. Bezüglich der Altersgruppen unterliegt die Zahl der durchgeführten Tests insbesondere in der Altersgruppe der > 80-Jährigen, als eine im Rahmen dieser Pandemie besonders betroffene Gruppe, relativ wenigen Schwankungen. Der auffällige Rückgang der Anzahl der durchgeführten Tests in allen Altersgruppen in der 52. und 53. Kalenderwoche kann mit dem veränderten Testverhalten um die Weihnachtsfeiertage erklärt werden.

**Figure 4:**
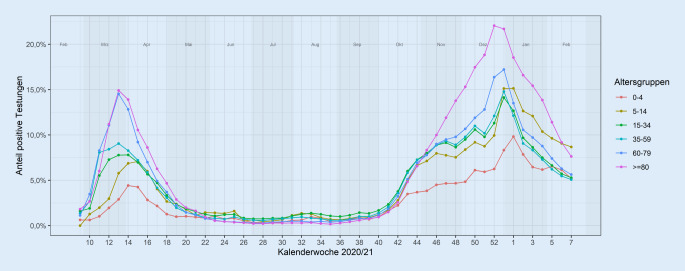

Abb. 4 zeigt den Anteil der positiven Testungen nach Altersgruppen und KW. Die größere Varianz der Positivanteile zwischen den Altersgruppen zu Beginn der Pandemie (Februar bis April 2020) im Vergleich zu den nachfolgenden Monaten (Mai bis September) ist am ehesten durch die geringeren Testzahlen in den ersten Wochen der Pandemie zu erklären. Ab Oktober 2020 lassen sich deutlich steigende Anteile positiver Testungen in allen Altersgruppen sehen. Die höchsten Anteile positiver Testungen mit bis zu 24 % finden sich in der Altersgruppe der > 80-Jährigen, bei einer relativ konstanten Anzahl durchgeführter Testungen in dieser Altersgruppe (Abb. 3).

Daten zu serologischer Diagnostik liegen aktuell aus 37 Laboren vor, die Grundlage weiterer wissenschaftlicher Auswertungen sind. In Zusammenarbeit mit den Laboren werden weitergehende Anpassungen der laborbasierten Surveillance SARS-CoV‑2 angestrebt, um z. B. auch die Dynamik des Auftretens von SARS-CoV-2-Varianten differenziert abbilden zu können.

Bisher nicht veröffentlichte Auswertungen konnten zeigen, dass mit der laborbasierten Surveillance SARS-CoV‑2 etwa 30–40 % aller bundesweit durchgeführten Testungen erfasst werden.

Eine Limitation ist die regional sehr unterschiedliche Abdeckung und damit die Repräsentativität regionaler Daten auf Ebene der Bundesländer und Landkreise. Eine weitere Limitation der laborbasierten Surveillance SARS-CoV‑2 sind die fehlenden klinischen Angaben zu den positiv getesteten Personen. Zwischen symptomatischen und asymptomatischen Personen kann nicht unterschieden werden, da die Surveillance SARS-CoV‑2 eine laborbasierte Surveillance ist und klinische Angaben zur Person im Labor nicht vorliegen.

### Abrechnungsdaten der Kassenärztlichen Vereinigungen

Eine weitere Datenquelle zur Bewertung der Testzahlen bilden Abrechnungsdaten der Kassenärztlichen Vereinigungen in Deutschland. Hierzu werden abgerechnete Leistungen von SARS-CoV-2-Tests aus dem niedergelassenen Bereich ausgewertet. Neben der genaueren Abgrenzung eines reellen Anstiegs von Infizierten gegenüber einem generellen Anstieg der Testraten könnten hier für einzelne Bundesländer mithilfe zusätzlicher patienten- und wohnortbezogener Informationen stratifizierte epidemiologische Auswertungen vorgenommen und somit weitere Erkenntnisse gewonnen werden. Auch eine Einschätzung der abgerechneten Mittel, die für Testungen und die Betreuung von Verdachtsfällen im niedergelassenen Bereich aufgewendet wurden, ist in diesem Zusammenhang möglich.

Seit dem 01.02.2020 ist die Testung auf eine Infektion mit dem neuartigen Coronavirus (SARS-CoV-2) eine über die Krankenversicherungen in Deutschland abrechenbare Leistung [13, 14]. Zusätzlich ist eine ärztliche Betreuung bei klinischem Verdacht gemäß der Falldefinition des RKI auf eine Infektion mit SARS-CoV‑2 oder einer nachgewiesenen Infektion bei der Abrechnung anzugeben [15]. Die von den Kassenärztlichen Vereinigungen bereitgestellten Abrechnungsdaten liefern tagesgenaue Informationen zur Häufigkeit der jeweiligen Abrechnungsziffer – d. h. zur Anzahl der durchgeführten Testungen und zur Anzahl der ärztlichen Untersuchungen eines (Verdachts‑)Falls. Außerdem liefern sie Informationen zur Anzahl unterschiedlicher Patienten, indem diese nur einmalig am Tag der ersten Abrechnung gezählt werden. Somit sind Rückschlüsse auf Mehrfachtestungen und -betreuungen möglich.

Die vorliegenden Daten erfassen ausschließlich den Zeitraum der KW 5 bis 13 des ersten Quartals 2020 und werden sukzessiv um Daten weiterer Abrechnungsquartale erweitert. Darüber hinaus werden ergänzende Abfragen durchgeführt, da es sich aktuell teilweise noch um unvollständige und nicht komplett durch die Kassenärztlichen Vereinigungen geprüfte Rohdaten handelt.

Tab. 1 zeigt Ergebnisse einer ersten Auswertung der Gesamtzahlen von abgerechneten Tests und Fällen bundesweit im niedergelassenen Bereich für gesetzlich krankenversicherte Personen. Weiterhin werden die Kosten der abgerechneten Tests angegeben, die zum dargestellten Zeitpunkt 59,00 € pro Test betrugen.

**Table 1:** 

KW	Gesamtzahl Tests^a^	Anzahl Patienten unter abgerechneten Tests	Gesamtzahl betreuter (Verdachts‑) Fälle^a^	Anzahl Patienten unter betreuten (Verdachts‑) Fällen	Gesamtkosten der abgerechneten Tests in €
*5*	204	192	3732	3669	12.036
*6*	353	312	6385	6052	20.827
*7*	216	180	6249	5903	12.744
*8*	306	275	5638	5287	18.054
*9*	12.157	11.174	35.491	31.747	717.263
*10*	29.388	27.743	72.529	60.502	1.733.892
*11*	100.249	95.653	261.108	215.910	5.914.691
*12*	180.431	171.871	377.513	290.592	10.645.429
*13*	178.989	166.821	321.382	234.880	10.560.351

^a^Inkl. Mehrfachtestungen bzw. Mehrfachbetreuungen von COVID-19-Verdachtsfällen

## Diskussion

Die Nutzung verschiedener Datenquellen für SARS-CoV-2-Labortestungen in Deutschland hat es ermöglicht, bereits früh in der Pandemie Daten und Auswertungen für die Beurteilung und Steuerung der Lage zur Verfügung zu stellen. Die Anpassungen aller Systeme in teilweise hoher Geschwindigkeit sind als Reaktion auf das hochdynamische Geschehen sowohl hinsichtlich der Patientenzahlen und durchgeführten Testungen als auch hinsichtlich der sich rasch entwickelnden diagnostischen Möglichkeiten zu verstehen. Mit der gesetzlichen Verankerung der Übermittlungspflicht für Labore nach § 7 IfSG wird der besonderen Bedeutung der mikrobiologischen Diagnostik und der Labor-Surveillance Rechnung getragen. Tab. 2 gibt einen Überblick über die Datensysteme, die für eine Gesamterfassung von COVID-19-Testzahlen und weiteren relevanten Parametern bezüglich der Pandemie herangezogen werden können.

**Table 2:** 

	Testlaborabfrage	Laborbasierte Surveillance SARS-CoV‑2	Abrechnungsdaten der Kassenärztlichen Vereinigungen
*Datenquellen*	Universitätskliniken, Forschungseinrichtungen, klinische und ambulante Labore	Labore, die Teil der ARS-Surveillance sind	Kassenärztliche Vereinigungen der Bundesländer
*Art der Erfassung/Übermittlung*	Online/VOXCO	Direkt aus dem Labor-Informationssystem (LIS) oder über Schnittstelle zu kommerzieller Statistiksoftware	Direkte Datenbereitstellung
*Frequenz der Erfassung*	Wöchentlich	Täglich	Quartalsweise
*Granularität der Daten*	Wochenaktuell	Tagesaktuell	Tagesaktuell
*Zuordnung positive/negative Befunde möglich?*	Ja	Ja	Nein
*Zusätzliche Parameter*	Aktuelle Kapazität der Labore zur Testung auf SARS-CoV‑2	Alter	*Gesamtzahlen an*
		Geschlecht	SARS-COV-2-Tests
		Regionale Zuordnung der Einsender	Anzahl getesteter Patienten
		Typ und Fachbereich der Einsender (Krankenhaus, Arztpraxis)	Betreuungen von COVID-19-(Verdachts‑)Fällen
		Sammelkategorie für Einsender wie Drive-in-Stationen, Testzentren, fremdeinsendende Labore	*Perspektivisch:* regionale und personenbezogene Zuordnung (Alter, Geschlecht, Wohnort, ICD-10-Diagnose, Diagnosesicherheit)
		Ergebnisse serologischer Diagnostik	
*Limitationen*	Freiwillige Teilnahme, keine Vollständigkeit der Testzahlen, keine Information über Anzahl der Labore, die SARS-CoV-2-Diagnostik durchführen	Freiwillige Teilnahme, Daten regional nicht immer repräsentativ	Aktuell: fehlende Zuordnung zum Wohnsitz der Patienten, keine Angaben zu Privatversicherten mit SARS-CoV-2-Tests möglich; keine Angaben zu durchgeführten Tests in Krankenhäusern, bei Gesundheitsämtern und Betriebsärzten

Eine Auswertung zu den in den Laboren durchgeführten PCR-Testungen zeigte, dass alle Labore ihre Testkapazitäten erhöhen konnten und der Hauptanteil der PCR-Testungen in Laboren mit weniger als 25.000 Testungen pro Woche erfolgt [16]. Die Rückmeldung der Labore hinsichtlich Auslastung der Kapazitäten und des daraus resultierenden Probenrückstaus hat zu einer Änderung der Testkriterien ab KW 45/2020 geführt. Dies hat zu einer Entlastung der Labore beigetragen und die durch die Überlastung bedingten verlängerten Bearbeitungszeiten wieder verkürzt. Gleichzeitig erreichte der Anteil der positiven Tests in KW 53/2020 mit ca. 15 % einen bisherigen Peak. Bei einem hohen Positivenanteil und gleichzeitig hohen Fallzahlen muss von einer starken Untererfassung ausgegangen werden. Gleichzeitig wird in Deutschland seit dem Jahreswechsel 2020/2021 trotz wieder erweiterter Testkriterien deutlich weniger getestet als in den Wochen zuvor. Dies kann auch durch den zunehmenden Einsatz von Antigenschnelltests bedingt sein. Da der Umfang der Anwendung dieser Tests nicht bekannt ist, kann der Einfluss auf die Anzahl der durchgeführten PCR-Tests nicht abgeschätzt werden.

Da es sich bei der RKI-Testzahlerfassung um eine freiwillige Übermittlung der Daten durch die Labore handelt, stellt diese keine Vollerfassung der durchgeführten PCR-Tests auf SARS-CoV‑2 dar. Durch die hohe Beteiligung der in mehreren Laborverbünden zusammengeschlossenen Labore und die Erfassung der Zahlen aller in der RKI-Testzahlerfassung genutzten Systeme ist eine weitgehende Repräsentativität auf Bundesebene gegeben. Dies ist auf Bundeslandebene und kleineren Ebenen jedoch nicht immer der Fall. Bei der Angabe des Positivenanteils pro KW ist zu beachten, dass der Anteil der positiven Testungen in den einzelnen Laboren von der Zusammensetzung der untersuchten Proben beeinflusst werden (Untersuchung von Ausbruchsproben, Reihentestung) und dementsprechend Schwankungen unterliegen kann [16].

Aufgrund der quartalsweisen Abrechnungsstrukturen können die Abrechnungsdaten der Kassenärztlichen Vereinigungen ausschließlich quartalsweise und nicht in kürzeren Perioden fortgeschrieben werden. Diese zeitlich eingeschränkte Datenverfügbarkeit steht einer zeitnahen Bewertung der Test- und Fallzahlen entgegen. Eine weitere Limitation ist die fehlende direkte Zuordnung zum Wohnsitz der Patienten: Es findet lediglich eine Zuordnung zur abrechnenden KV-Region statt. Für die Erkrankungsfälle ist dies weniger problematisch als für die abgerechneten Tests. Laborpraxen rechnen ihre Leistungen im Bereich ihrer KV-Region (Standort des Labors/Sitz der Praxis) ab, aber erhalten über die KV-Grenzen hinaus Proben. Daher haben diese Daten nur einen schwachen regionalen Bezug zum Patienten.

## Fazit

Die Erhebung von Daten zu Testungen von SARS-CoV‑2 durch verschiedene Surveillance-Systeme und Instrumente stellt für alle an der Datenbereitstellung und -verarbeitung Beteiligten einen hohen personellen und technischen Aufwand dar. Die Daten aus allen Systemen werden zum einen für bestimmte Fragestellungen am RKI zusammengeführt und abgeglichen, zum anderen hat jedes System Stärken für die Beantwortung spezifischer Fragen. Somit stellen die sich ergänzenden Surveillance-Systeme eine Bereicherung und wichtige Säule für die Beurteilung und Steuerung der Maßnahmen zur Pandemiebewältigung dar. Eine Integration der negativen SARS-CoV-2-PCR-Tests in die elektronische Meldepflicht würde dies erleichtern und gleichzeitig ein umfassenderes Bild der Umsetzung der Teststrategie ermöglichen.
